# Personal Experience with Puerperal Fever1Read at the 36th annual meeting of the Medical Association of Central New York, held at Auburn, September 22, 1903.

**Published:** 1903-12

**Authors:** Charles E. Congdon

**Affiliations:** Buffalo, N. Y.


### Personal Experience With Puerperal Fever.[1]

By CHARLES E. CONGDON, M. D., Buffalo, N. Y.

IT IS not my object to present a review of typical puerperal infection, inflammation of the ovaries, tubes and the contiguous parts or their many complications with the confusing symptoms. I will, however, give some of the cardinal points in regard to diagnosis and treatment as a result of my own experience. It is an established fact that our ideas in regard to this disease have met fundamental changes since Oliver Wendell Holmes wrote his masterly essay in 1843. We now know that this condition is due to a bacterial infection and not to retained secundines or blood clots per se, the origin of which has always been more or less hypothetic.

In accordance with the virulence of the bacteria the symptoms become mild or severe, acute or chronic. The necessary factors for the presence of such an infection are an invasion of bacteria,

1. Read at the 36th annual meeting of the Medical Association of Central New York, held at Auburn, September 22, 1903.

either from the blood, the intestinal tract or from without. In the normal condition the vagina is sterile and the uterine cavity is free from bacteria, but the vulva is not. Whenever an infection atrium is established by lacerations of the cervix uteri or any of the soft parts, then there is always a favorable opportunity afforded for a bacterial invasion followed by various pathologic changes in the vagina, uterus, adnexa or contiguous parts. Most frequently the invasion is plainly from without as the lesion begins in the pelvic organs and remains confined to them and the contiguous parts in nearly all cases. Less frequently and less certainly, from the blood and intestinal tract.

There is little need for me to remind you of the various microorganisms found to be the invading germs or of the method of determining the principal or various microbic invasions found in the uterine cavity. Döderlein's tube is the means universally employed, yet in the light of our present knowledge, the scope of its usefulness is not so great as at first hoped for; though this is not due to the method, but to the inability to determine the virulence of the bacteria. Permit me to quote Fehling's own words. He says:

> The attempt to divide the various forms of puerperal infection bacteriologically cannot be considered thus far as successful; this much is certain, that in numerous cases that have run a normal course streptococci and staphylococci have been found in the uterine cavity; and further, in the severe forms of infection sometimes streptococci, sometimes staphylococci, at other times bacteria coli communis and other bacteria or mixtures of these are found, thus demonstrating that the attempt to make a ready and positive diagnosis from a bacteriological examination thus far has been a failure.

As yet we know that an infection takes place, but to be able accurately to determine the virulence of the infection or the infection atrium is only problematic. And in view of the fact that a bacteriologic examination is to be relied upon only in exceptional cases, we are brought to the question of, what is a reliable means of arriving at a positive diagnosis? What tissues are involved? What are the limitations of the infection? In answer to these questions, permit me briefly to cite the diagnosis and treatment of a sufficient number of the 22 cases which I have personally treated, to try and make this point somewhat clear.

In one patient there were rapid pulse, high temperature, profuse perspiration, anxious expression with a history of a spontaneous delivery some days previous to my seeing her, in which the placenta was not delivered for three or four days, although the patient attempted its removal herself; then a physician was called who delivered it. Two days later she developed pronounced puerperal sepsis; uterus was markedly enlarged, no ascertainable lesion. As soon as she was removed to the hospital it was found that there was a large collection of pus in the uterine cavity; digital exploration revealed the absence of placental tissue or other lesion which might account for her condition. This was diagnosticated as an endometritis without involvement of other organs or tissues. The treatment was continuous irrigation of the uterine cavity for several days, and later it was given at intervals until her temperature became normal. She made a perfect recovery. This was a patient of Dr. Seidler, who referred the case to me.

The next case was delivered by a physician unknown to me. Three days following labor she developed sepsis and her condition was so alarming, that death seemed imminent; the physician was discharged and Dr. Clements was called, who referred the case to me. She was immediately removed to the hospital. The only ascertainable lesions were extensive lacerations of the vagina and vulva, which had become infected. Continuous irrigation effected a cure.

In another case, upon examination no special lesion was found, however she had a subnormal temperature, rapid pulse, frequent effortless emesis, flattened abdomen; in other words, she had general septic peritonitis, as the postmortem examination soon proved, the infection having taken place through a trivial cervical laceration.

Another case with abdominal distention and rigidity more noticeable in the lower part, constipated, with uterus movable, had high temperature, rapid pulse, and great exhaustion. The diagnosis was made of uterine abscess without involvement of other organs. At the abdominal section, this was verified. A number of abscess cavities were found immediately underlying the peritoneum and on one side beginning to extend to the broad ligament. The entire body of the uterus was very friable. The hysterectomy did not prolong her life as she died of shock. This case and the following were patients of Dr. Woodbury.

In still another case the patient entered the hospital with the high temperature and rapid pulse characteristic of this condition. Abdominal inspection and palpation were negative; vaginal and uterine examinations were negative except for a thickening of the broad ligament, especially noticeable on the left side. On opening the abdomen this proved to be a case of thrombosed veins and dilated lymphatics of the broad ligaments. Removal of these with the uterus effected a cure.

In yet another case, that had in connection with rapid pulse and high temperature and delirium, a copious diarrhea of a very offensive odor, without pain or special tenderness, abdominal palpation and inspection elicited slight tympanites and muscular rigidity in the lower part of the abdomen. Vaginal examination, negative. Upon section of the posterior vaginal wall peritonitis was evident, and an ounce or more of pus was evacuated, which came from the region of the left ovary and a plastic exudate was everywhere present especially on the left side, extending to the flank. This patient made a very quick and easy recovery.

The next patient entered the hospital with a history of having had a miscarriage three days previously. She had accelerated pulse, a slightly elevated temperature and was still flowing. After a most careful preparation she was cureted and some placental tissue removed. Digital examination of the uterine cavity failed to disclose anything unusual, the ovaries and tubes also being normal. Three days later her pulse and temperature began to ascend and an examination revealed an enlargement of the left ovary, which was removed through an opening in the posterior cul-de-sac. The ovary was about the size of a large English walnut and contained nearly a dram of pus, the tube was not involved or removed. The patient's temperature became normal and remained so. A perfect recovery followed.

In another case of this condition, in which there was high temperature, rapid, feeble pulse, and great exhaustion, abdominal tympanites was present, but especially marked in the lower abdomen. Vaginal examination failed to reveal anything unusual. Upon opening the abdomen a considerable amount of serosanguinous pus was found; the intestines were injected and darkened, plastic lymph was present, while both ovaries and tubes were dark and necrotic. However, they were not removed, but a drain was placed and the patient made an uninterrupted recovery.

Of the 22 cases which I have had, of the 7 which were not subjected to operative procedures, 2 were properly treated, the other 5 were treated by the methods almost universally employed today, such as curetment, irrigation, intravenous or subcutaneous medication, douches, both medicated and nonmedicated, intrauterine medication, direct application of various forms of salves in the uterus, vagina, over the abdomen and in the axilla.

The remaining 15 were treated surgically as follows: 3 abdominal hysterectomies, 1 recovery, 2 deaths; 1 vaginal hysterectomy; this case also succumbed. Of 5 abdominal sections with drainage,

1 died, 4 recovered. In 5 vaginal sections, all recovered. One patient treated by the combined method,—vaginal and abdominal section,—recovered. Of these 15 cases receiving operative treatment 4 died, 11 recovered. The 7 not receiving operative treatment, 5 died, and 2 recovered. Thus far the treatment of puerperal infection has been very unsatisfactory for the reason that it has been treated as a special disease and not as a variety of conditions the result of infection, and my experience leads me to pay special attention to the exact pathological condition present, to determine if the infection has gained entrance through lacerations of the soft parts,—the uterus itself and especially the cervix,—or through the corpus luteum. To say that the curet should never be used does not coincide with my experience, but in most cases I believe it better to err in not using it.

Irrigation is of undoubted value in certain cases, but it will be noted that it proved useful in but 2 of the 22 cases reported. A careful search for and inspection of lacerations, the discovery of the probable infection atrium and its appropriate treatment will avoid the often fatal error of using the curet, the douche or the scalpel unadvisedly, and when it is definitely determined that the uterine cavity contains no secundines, or is not the seat of suppurating fibroids and that the cervix, vagina, or vulva is not the probable seat of the infection, and when intestinal toxemia is eliminated, then and then only should section be thought of. An early recognition and removal of an ovarian abscess, as in my case 22, is the ideal treatment.

Hysterectomy has been so fatal not only in my hands but in others, that it is only to be thought of in very exceptional cases. Section and drainage of the pelvis, either through the vagina or from above, when the inflammatory process has extended to the contiguous parts, the ovaries, tubes or peritoneum, whether accompanied by pus formation or not, gives immediate and permanent relief; where the inflammatory process has rapidly formed in the cellular tissue of the broad ligaments, vaginal section should be made into the peritoneum, and then by separating the tissues these collections may be drained; if complicated by pelvic abscess the peritoneum itself may be opened. In the forepart of this paper the assertion is made that the inflammatory process is confined to the pelvis in the majority of cases, but if this condition is permitted to extend unchecked there is no positive assurance that it may not extend to any organ or tissue of the body; therefore prompt attention and quick decision will avoid complications.

1034 Jefferson Street.

